# Prognostic value of extravascular lung water assessed with lung ultrasound score by chest sonography in patients with acute respiratory distress syndrome

**DOI:** 10.1186/s12890-015-0091-2

**Published:** 2015-08-23

**Authors:** Zhen Zhao, Li Jiang, Xiuming Xi, Qi Jiang, Bo Zhu, Meiping Wang, Jin Xing, Dan Zhang

**Affiliations:** Intensive Care Unit, Fuxing Hospital affiliated with Capital Medical University, Beijing, 100038 China; Department of Ultrasonography, Fuxing Hospital affiliated with Capital Medical University, Beijing, 100038 China

## Abstract

**Background:**

The prognostic value of extravascular lung water indices (EVLWI) has been widely investigated, which is determined by lung ultrasound B-lines. However, the clinical value of lung ultrasound B-lines for determining prognosis/intensive care unit (ICU) outcomes in patients with acute respiratory distress syndrome (ARDS) has been rarely reported.

**Methods:**

Twenty-one ARDS patients admitted to the ICU of Fu Xing Hospital underwent both lung ultrasonography and pulse index continuous cardiac output (PiCCO) monitoring on the first, second, and third days after diagnosis. The correlation between lung ultrasound score (LUS) and EVLWI measured by the PiCCO system was investigated. The prognostic clinical value of lung ultrasonography in ARDS patients was explored. Chest ultrasound was performed using the 12 regions method. The comprehensive score of lung ultrasound was determined according to the level of lung aeration.

**Results:**

With ICU mortality as the end point, 21 patients were divided into a survivor group (8 patients, 39.1 %) and a non-survivor group (13 patients, 61.9 %). Significant positive linear correlations were found between LUS and EVLWI, including predicted body weight (*r*^2^ = 0.906), sequential organ failure assessment score (*r*^2^ = 0.815), lung injury score (*r*^2^ = 0.361), and PaO_2_/FiO_2_ (*r*^2^ = 0.472). Significantly different LUSs were found between the non-survivor and survivor groups (*F* = 77.64, *P* <0.01) by repeated-measures analysis of variance. There were no significant differences between the two groups on different days. The areas under the receiver operating characteristic curves of LUS and EVLW measured by PiCCO were 0.846 (*P* < 0.01) and 0.918 (*P* < 0.01), respectively. The cut-off of LUS for prognosis prediction was 16.5.

**Conclusions:**

Lung ultrasonography is a non-invasive, economic, simple, user-friendly, and radiation-free bedside method for predicting the prognosis of ARDS patients. Early measurement of LUS is a better prognostic indicator in patients with ARDS.

## Background

Acute respiratory distress syndrome (ARDS) is a common complication in critically ill patients. In the intensive care unit (ICU), 19 % of patients with mechanical ventilation are diagnosed with ARDS, which is characterized by increased extravascular lung water (EVLW) and refractory hypoxemia, with mortality rates as high as 32–65 % [1]. Diagnostic methods, such as physical examination, bedside X-ray chest film, and computed tomography (CT), provide a basic clinical evaluation of EVLW in severe cases, and they are useful for assessing lung involvement in ARDS. However, the accuracy of the former two methods is less than optimal [2]. CT scans increase safety risks because they require patient transport. Pulse index continuous cardiac output (PiCCO) technology is a highly accurate quantitative method for determination of EVLW, but it is invasive and may cause catheter-related infections [3]. Lung ultrasound is used to detect pulmonary edema in the early period, and ‘B-lines’ indicate loss of lung aeration, which may be caused by an increase in EVLW [4]. However, some researchers have introduced LUS, according to the different pulmonary ultrasonography of ARDS patients, to determine EVLW semi-quantitatively [5].

In the present study, the correlation between lung ultrasound and PiCCO monitoring was investigated to determine EVLW and evaluate the value of these two examinations in predicting ICU prognosis of ARDS patients. This economic and noninvasive bedside examination is expected to be used in the prognostic evaluation of ARDS patients.

## Methods

### Patients

Criteria for inclusion: (1) ARDS patients admitted to the ICU of Fu Xing Hospital affiliated with the Capital University of Medical Sciences from June 2012 to May 2013; (2) Patients meeting the diagnostic criteria of ARDS [6].

Criteria for exclusion:(1) Patients who spent ≤ 24 h in the ICU; (2) Patients whose mechanical ventilation time was ≤ 24 h; (3) Patients with severe hemodynamic instability who were unable to safely change body positions; (4) Patients with severely deformed chest cage or subcutaneous emphysema who were unfit for lung ultrasound; (5) Patients who agreed to limit or withdraw life support treatment during hospitalization;(6) Patients whose family did not sign the informed consent.

### Data collection

All patients were confirmed to have ARDS by chest CT on the first day of admission to the hospital. Patients were studied on the first, second, and third day after diagnosis of ARDS by lung ultrasound, bedside X-ray film, and PiCCO monitoring. Lung ultrasound was performed immediately after PiCCO monitoring.

Epidemiological data were collected, including sex, age, height, ideal weight, ARDS etiology, incidence period, and ICU outcome. The sequential organ failure assessment (SOFA) scores and lung injury score (LIS) scores of the patients were recorded every day. Parameters of mechanical ventilation were also collected every day, including tidal volume index, positive end-expiratory pressure (PEEP), static respiratory system compliance (Crs), and PaO_2_/FiO_2_ (P/F).

The study was approved by the Ethics Committee of Beijing Fuxing Hospital, Capital Medical University and met all ethical requirements. All participants in both groups voluntarily joined this study and provided written informed consent.

### EVLW assessment

#### Lung ultrasound

**Fig. 1:**
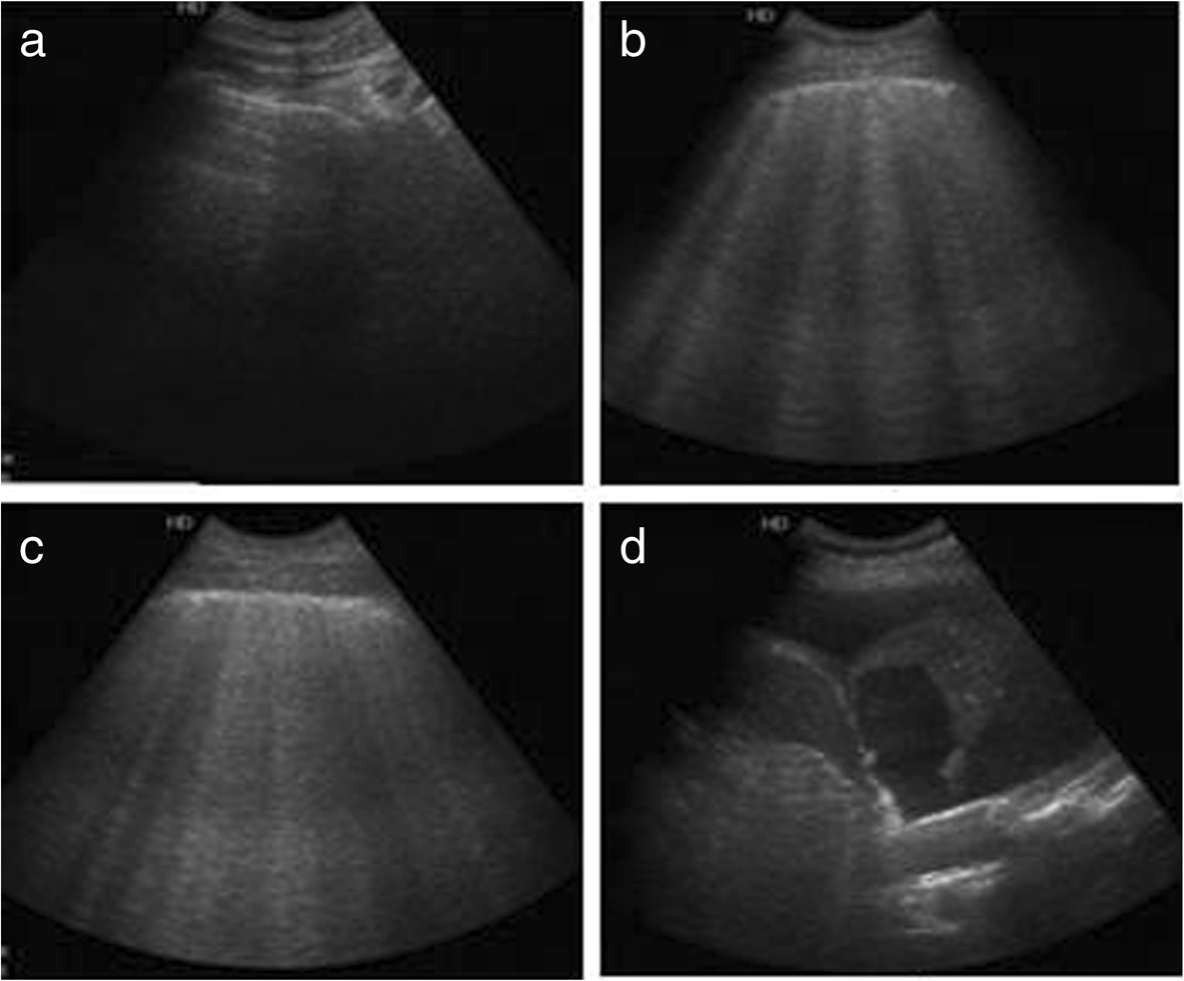
Four ultrasound patterns according to lung aeration. **a** normal aeration (N): presence of lung sliding with A lines or fewer than two isolated B lines; **b** moderate loss of lung aeration: multiple, well-defined B lines (B1 lines); **c** severe loss of lung aeration: multiple coalescent B lines (B2 lines); and **d** lung consolidation (C), the presence of a tissue pattern. For a given region of interest, points were allocated according to the worst ultrasound pattern observed: N = 0, B1 lines = 1, B2 lines = 2, C = 3

Lung ultrasound was performed to diagnose ARDS. A Philips C5 ultrasound system (frequency 5Hz; Philips Medical Systems, Suresnes, France) with an ordinary convex probe was used. Chest ultrasound was performed using the 12 regions method. All intercostal spaces of the upper and lower parts of the anterior, lateral, and posterior regions of the left and right chest walls were examined. Each region of interest was extensively examined. The worst ultrasound abnormality detected was considered to characterize the region being examined. Four ultrasound aeration patterns as shown in Fig. 1 were defined [5]: a. normal aeration (N): line sliding sign associated with respiratory movement or less than 3 B lines; b. moderate loss of lung aeration: a clear number of multiple visible B-lines with horizontal spacing between adjacent B lines ≤ 7 mm (B1 lines); c. severe loss of lung aeration: multiple B lines fused together that were difficult to count with horizontal spacing between adjacent B lines ≤ 3 mm, including’white lung’ (B2 lines); and d. pulmonary consolidation (C), hyperechoic lung tissue, accompanied by dynamic air bronchogram.

LUS was determined based on four lung ultrasonographs: N = 0, B1 = 1, B2 = 2, and C = 3. All patients underwent a lung ultrasound, and each of the 12 lung areas was examined. The final LUS of the patient was the sum of each regional ultrasound score (ranging from 0 to 36).

All lung ultrasound images were examined by two ultrasound doctors. Both doctors were blind to the clinical data of the patients and to the other doctor’s ultrasound diagnosis.

#### PiCCO monitoring

EVLW was measured using a PiCCO system (Pulsiocath PV8115; Pulsion Medical Systems, Feldkirchen, Germany). For each patient, after 15 ml of 0 °C 0.9 % saline bolus was injected into the central vein, artery temperature was detected using a temperature sensor in a femoral artery catheter, and EVLW was calculated. The final EVLW was the average of three consecutive injections [7]. Extravascular lung water index (EVLWI) was the surface distribution of extra-vascular lung water in a predicted body weight (PBW). Male: PBW(kg) = 0.91(height (cm) − 152.4) +50; female: PBW(kg) = 0.91 (height (cm) −152.4) +45.5 [8].

### Statistical analysis

With ICU mortality as the end point, all patients were divided into a survivor group and a non-survivor group. Normally distributed continuous quantitative data were described as means ± standard deviation (mean ± standard deviation (SD)). Normal continuous variables were compared using the *t* test, and non-normal continuous data were compared using the Mann-Whitney *U* test. One-way analysis of variance was used to compare more than two independent variables. To access ICU outcome, a linear regression model was built that included LUS and other ARDS prognostic indices. Additionally, scatter grams (GraphPad Prism5) and receiver operating characteristic (ROC) analyses were performed.

## Results

Twenty-nine ARDS patients were selected from June 2012 to March 2013. Eight of these patients matched the exclusion criteria, including three patients who died within 24 h, two patients who were unable to complete the lung ultrasound, and three patients who signed restrictions or asked for withdrawal from life support. The clinical characteristics of 21 patients with ARDS are shown in Table 1. The average age of all patients was 78 ± 6 years, their average height was 169 ± 8 cm, and their average weight was 66 ± 7 kg. ARDS was caused by pneumonia in 13 patients (62 %), aspiration in three patients (14 %), cardiopulmonary resuscitation in another three patients (14 %), and trauma in one patient (5 %). Fifteen patients (72 %) in the ICU were diagnosed with ARDS within 48 h. Thirteen patients (62 %) died in the ICU.

**Table 1:** Clinical characteristics of 21 patients with acute respiratory distress syndrome

Variable	Mean ± SD
Age (years)	78 ± 6
Gender	
Male	14 (67 %)
Female	7 (33 %)
Height (cm)	169 ± 8
PBW (kg)	66 ± 7
CRX quadrants	
3	9 (43 %)
4	12 (57 %)
Etiology of ARDS	
Pneumonia	13 (62 %)
Aspiration	3 (14 %)
Sepsis	3 (14 %)
CPR	1 (5 %)
Trauma	1 (5 %)
Onset	
<48 h	15 (72 %)
>48 h	6 (28 %)
ICU mortality	
Survival	13 (62 %)
Non-survival	8 (38 %)

*PBW* predicted body weight, *CRX* chest X-ray, *CPR* cardio pulmonary resuscitation

**Table 2:** The characteristics of patients on day 1

	All	Survivors	Non-survivors	*p*
	(*n* = 21)	(*n* = 8)	(*n* = 13)	
Age (years)	78 ± 6	76 ± 3	80 ± 2	0.228
Male (n (%))	14 (67)	5 (63)	9 (69)	<0.01
Height (cm)	169 ± 8	170 ± 9	169 ± 8	0.866
PBW (kg)	66 ± 7	66 ± 8	65 ± 7	0.866
SOFA	13 ± 2.6	10 ± 2	15 ± 2	<0.01
PEEP (cm H_2_O)	10 ± 3	8 ± 2	11 ± 3	0.036
Crs (mL/cm H_2_O)	34 ± 8	39 ± 8	31 ± 6	0.013
P/F (mmHg)	160 ± 42	184 ± 23	145 ± 45	0.019
VT (ml/kg PBW)	6.6 ± 0.5	6.4 ± 0.5	6.7 ± 0.5	0.109
LIS	2.8 ± 0.4	2.4 ± 0.3	3 ± 0.3	0.001
LUS	18.1 ± 5.3	15 ± 5	20 ± 5	0.022
EVLWI	16.8 ± 4.4	13 ± 3.6	19 ± 3	<0.01

*PBW* predicted body weight, *SOFA* sequential organ failure assessment score, *PEEP* positive end expiratory pressure, *Crs* static respiratory system compliance, *P/F* PaO2/FiO2, *VT* tidal volume, *LIS* lung injury score, *LUS* lung ultrasound score, *EVLWI* extra-vascular lung water indexed to PBW

**Fig. 2:**
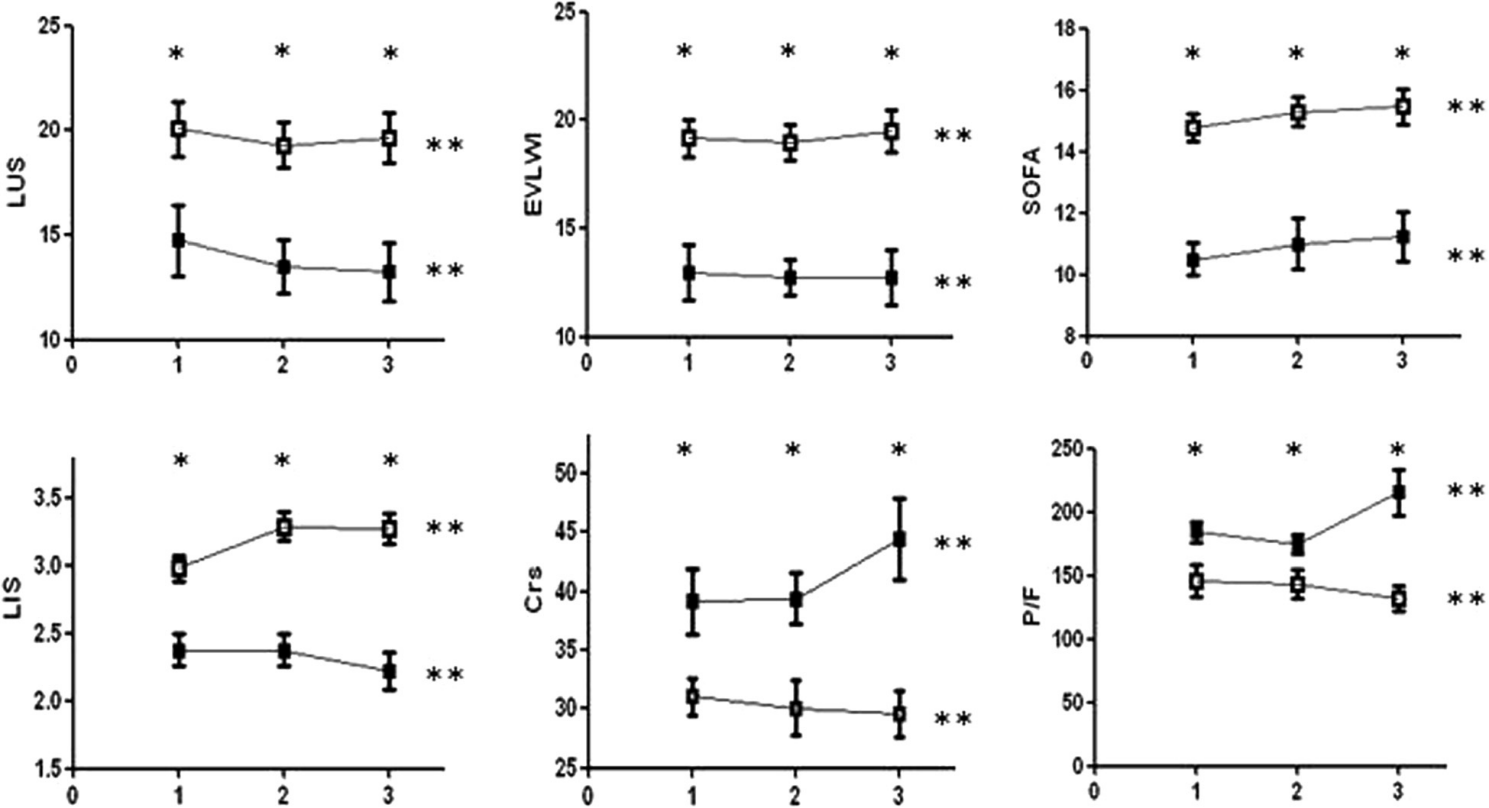
Lung dysfunction in the first 3 days following diagnosis of acute respiratory distress syndrome. Non-survivors are represented by unfilled squares and survivors are represented by filled ones. Plots show the means of values of 3 days vs. time for the following: lung ultrasound score (LUS); EVLWI: extra-vascular lung water indexed to predicted body weight (EVLWI) measured by pulse index continuous cardiac output (PiCCO); sequential organ failure assessment (SOFA); lung injury score (LIS); static respiratory system compliance (Crs); PaO_2_/FiO_2_(P/F). *: *P* < 0.05, significant differences were found between the non-survivor and survivor groups. **: *P* > 0.05, no significant differences were found between the two groups on different days

**Fig. 3:**
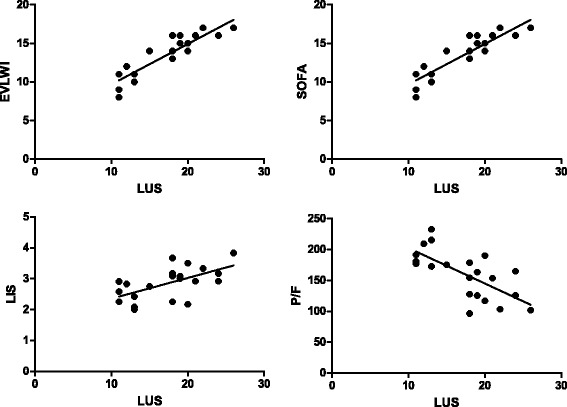
Scatterplots demonstrating the correlation between lung ultrasound score (LUS) and lung dysfunction. The graphs show pooled average data for all 3 days for survivors and non-survivors. EVLWI, extravascular lung water indexed to predicted body weight; P/F PaO_2_/FiO_2_; SOFA, sequential organ failure assessment score; LIS, lung injury score. Significant positive linear correlations were found between LUS and EVLWI, SOFA, LIS, P/F (r^2^ = 0.906, 0.815, 0.361, 0.472, P < 0.01)

As shown in Table 2, the LUS of ARDS patients in the non-survivor group was significantly higher than that in the survivor group (20 ± 5*vs.* 15 ± 5, *P* = 0.022). EVLWI (16.8 ± 4.4 mL/kg), SOFA (15 ± 2), LIS (3 ± 0.3), and Crs (39 ± 8) in the non-survivor group were significantly higher than in the survivor group on the first day, while age, height, PBW, and tidal volume were not significantly different between the two groups. Moreover, Crs and P/F in the non-survivor group were significantly lower (34 ± 8 mL/cmH_2_O, 160 ± 42 mmHg) than those in the survivor group. LUS of the non-survivor group was markedly higher than in the survivor group in the first 3 days following diagnosis of acute respiratory distress syndrome, and the other examined indicators (EVLWI, SOFA, LIS, Crs, P/F) exhibited the same tendencies with the degree of disease (Fig. 2). The average LUS and the EVLWI, SOFA, P/F, and LIS on the third day were significantly correlated (*r*^*2*^ = 0.906,0.815,0.472,0.361, *P* < 0.01) (Fig. 3).

**Fig. 4:**
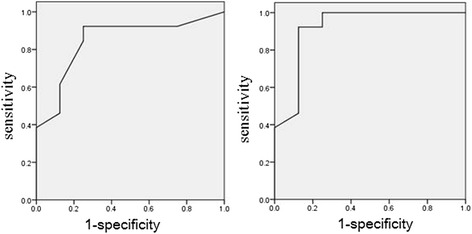
Receiver operating characteristic curves of lung ultrasound score (LUS) and extra-vascular lung water (EVLW) for mortality. The area under the curve (95 % confidence interval [CI]) was 0.846 ± 0.91 and 0.918 ± 0.72 for LUS and EVLW, respectively

Significant differences in LUS and EVLWI overall for 3 days were found between the non-survivor group and the survivor group (*F* = 11.82, 22.08, *P* < 0.01). The non-survivor group had significantly higher LUS and EVLWI values than the survivor group. The ROC curves of the average LUS and EVLWI values on the third day were valuable for evaluating clinical prognosis. There were no significant differences between the two groups in LUS or EVLWI on different days. The areas under the ROC curves of LUS and EVLW as determined by PiCCO were 0.846(*P* < 0.01) and 0.918(*P* < 0.01), respectively. The cut-off of LUS for prognosis prediction was 16.5 (Fig. 4).

## Discussion

Increased EVLW is the most important pathophysiological sign of ARDS. Additionally, the severity of pulmonary edema is closely related to the prognosis of ARDS patients. Several quantitative methods are clinically used to assess EVLW [9]. Bedside chest X-ray film is the simplest method but it is difficult to immediately detect changes in EVLW using this method. Although semi-quantitative determinations of EVLW have been made, the scores obtained were found to have a large respective divergence and to be inaccurate [10, 11]. CT is the gold standard for assessing EVLW. Changes in CT images in regard to pleural effusion, pulmonary interstitial syndrome, and pulmonary consolidation clearly confirm the diagnosis of ARDS. Moreover, chest CT software has been developed to accurately measure EVLW. However, to perform a CT scan, the patient has to be moved to the CT room. This movement is particularly dangerous, especially for patients with severe unstable hemodynamics, and it may even threaten the patient’s life [12, 13]. Recently, PiCCO technology has become widely used in clinics [14]. Kuzkovet al. found a positive linear correlation between EVLW and acute lung injury severity index (Crs, P/F, and LIS), and this correlation can help doctors to effectively characterize the clinical prognosis of patients [15]. PiCCO technology can provide a quantitative determination of EVLW, and EVLWI has emerged as a widely used method to evaluate the severity of pulmonary edema. However, PiCCO technology is expensive, and the procedure is invasive, therefore it puts patients at risk of infection because of the use of a catheter, and this limits its clinical application.

Lung ultrasonography has been widely used to assess EVLW clinically in recent years. Jambrik et al. determined the relationship between lung ultrasound and chest X-ray using PiCCO technology to assess EVLW [16]. To date, lung ultrasound has been applied in differential diagnosis of the acute respiratory failure etiology [17], in the EVLW assessment of hemodialysis (HD) patients [18, 19], even more in the extravascular lung water assessment of heart failure patients [20, 21], concluding that lung ultrasound is a relatively new method which has gained a growing acceptance as a bedside diagnostic tool to assess pulmonary interstitial fluid and alveolar oedema [22]. Also, lung ultrasound has been applied in the selection of the best PEEP of ARDS patients [23]. PEEP > 5cmH_2_O might aid in the prevention of alveolar collapse, improve the oxygenation state, and reduce ventilator-induced lung injury [24]. The strategy of low tidal volume and high PEEP ventilation could significantly reduce 28-day mortality in ARDS patients [25], increase the number of days of weaning success, and reduce the incidence of organ failure [26, 27]. However, the strategies of high PEEP ventilation and low PEEP ventilation did not show significant differences in regard to the prognosis of ARDS patients [28–30]. A meta-analysis showed that a high PEEP level could reduce the mortality of patients with moderate or severe ARDS (P/F ≤ 200 mmHg) but had no effect on the mortality of patients with mild ARDS [31].

Lung ultrasonography is rarely used in prognostic evaluation of ARDS patients. Frassi et al. divided patients with chest pain and dyspnea into mild, moderate, and severe groups based on the number of lung ultrasound comet tails (ULCs) and found a direct correlation between the number of ULCs and mortality rate [32]. Acute Physiology and Chronic Health Evaluation II and SOFA scores are good predictors of death risk in ARDS patients, while LIS and P/F have lower prognostic abilities [33–35]. Recently, EVLWI measured using PiCCO technology is being used with increasing frequency to assess ARDS prognosis. EVLWI is directly correlated with LIS, Crs, and, obviously, P/F [36–38]. Additionally, EVLWI of pediatric patients with acute respiratory distress could predict survival prognosis and mechanical ventilation duration [39]. A meta-analysis covering 670 patients in 11 studies of 9 countries showed that EVLWI was a better predictor of the mortality of severe patients [40].

In this study, LUS was closely related to several ARDS prognostic indices (EVLWI, LIS, Crs, and P/F) and was able to predict death risk and served as a diagnostic marker of ARDS. Early measurement of LUS is a better diagnostic indicator of acute lung injury than late measurement. Compared with other examinations, lung ultrasound is a non-invasive, economic, repeatable, simple, user-friendly, radiation-free bedside method for the prognosis of ARDS patients and for determination of the best treatment plan.

## Conclusions

In summary, significant positive correlations were found between LUS and EVLWI measured by PiCCO. Both were effective clinical examination tools for the evaluation of ICU outcome in ARDS patients. Lung ultrasound diagnosis is simple, quick, non-invasive, economic, and rather accurate and is recommended for clinical use.

## Abbreviations

ARDS: Acute respiratory distress syndrome
PiCCO: Pulse index continuous cardiac output
LUS: Lung ultrasound score
PBW: Predicted body weight
EVLW: Extra-vascular lung water
EVLWI: Extra-vascular lung water indexed to PBW
SOFA: Sequential organ failure assessment score
LIS: Lung injury score
P/F: PaO_2_/FiO_2_
ICU: Intensive care unit
PEEP: Positive end expiratory pressure
Crs: Static respiratory system compliance
